# Nowcasting daily minimum air and grass temperature

**DOI:** 10.1007/s00484-015-1017-7

**Published:** 2015-06-30

**Authors:** M. J. Savage

**Affiliations:** Agrometeorology Discipline, Soil–Plant–Atmosphere Continuum Research Unit, School of Agricultural, Earth and Environmental Sciences, University of KwaZulu-Natal, Pietermaritzburg, South Africa

**Keywords:** Diurnal temperature modelling, Early warning of minimum temperature, Frost, Sub-hourly temperatures, Surface temperature

## Abstract

Site-specific and accurate prediction of daily minimum air and grass temperatures, made available online several hours before their occurrence, would be of significant benefit to several economic sectors and for planning human activities. Site-specific and reasonably accurate nowcasts of daily minimum temperature several hours before its occurrence, using measured sub-hourly temperatures hours earlier in the morning as model inputs, was investigated. Various temperature models were tested for their ability to accurately nowcast daily minimum temperatures 2 or 4 h before sunrise. Temperature datasets used for the model nowcasts included sub-hourly grass and grass-surface (infrared) temperatures from one location in South Africa and air temperature from four subtropical sites varying in altitude (USA and South Africa) and from one site in central sub-Saharan Africa. Nowcast models used employed either exponential or square root functions to describe the rate of nighttime temperature decrease but inverted so as to determine the minimum temperature. The models were also applied in near real-time using an open web-based system to display the nowcasts. Extrapolation algorithms for the site-specific nowcasts were also implemented in a datalogger in an innovative and mathematically consistent manner. Comparison of model 1 (exponential) nowcasts vs measured daily minima air temperatures yielded root mean square errors (RMSEs) <1 °C for the 2-h ahead nowcasts. Model 2 (also exponential), for which a constant model coefficient (*b* = 2.2) was used, was usually slightly less accurate but still with RMSEs <1 °C. Use of model 3 (square root) yielded increased RMSEs for the 2-h ahead comparisons between nowcasted and measured daily minima air temperature, increasing to 1.4 °C for some sites. For all sites for all models, the comparisons for the 4-h ahead air temperature nowcasts generally yielded increased RMSEs, <2.1 °C. Comparisons for all model nowcasts of the daily grass and grass-surface minima yielded increased RMSEs compared to those for air temperature at 2 m. The sufficiently small RMSEs using the 2-h ahead nowcasts of the air temperature minimum, for the exponential model, demonstrate that the methodology used may be applied operationally but with increased errors for grass minimum temperature and the 4-h nowcasts.

## Introduction

While national weather services may forecast daily minimum temperature several days in advance, the predictions are usually not sufficiently site specific and do not take vegetation effects, for example, into account (Wu et al. 2011). Routine, timely, reasonably accurate and site-specific nowcast of temperature conditions several hours before the daily minimum temperature, particularly on a frost morning for example, is investigated. Technological advances over several decades now allow sub-hourly temperature measurements, on-board datalogger computation and data telecommunication capability and therefore allow opportunities for nowcasting. Early nowcasting and early warning of daily minimum air temperature would be of great economic and operational benefit, for example, to animal and crop production, as well as to manufacturing enterprises and the lay public for planning of their human activities. City, district, or regional weather predictions are often not sufficiently accurate in nowcasting near-surface daily minimum temperatures and cannot be used with confidence for specific sites with different topography, altitude, soil-surface cover, etc.

The terminology in relation to the subdivision of very short-range weather forecasts into weather nowcasting and nearcasting is not consistent. Glickman (2000) refers to nowcasting as a short-term weather forecast, generally for the next few hours, also stating that the US National Weather Service specifies <3 h, but that up to 6 h has also been used. Garcia et al. (2010, Fig. 7.3) state a time period of <10^4^ s—or just <3 h—with Das et al. (2010, Table 5.1) stating <2 h. For the purposes of this work, the term nowcasting will imply time periods <4 h. However, the time period for data used for nowcasting estimations was 6 h ahead of current time. This investigation, similar to complex event processing (CEP) used for the observation and management of business processes (Janiesch et al. 2012), proposes a dynamic measurement, open web-based early nowcasting and control system based on sub-hourly and site-specific temperature measurements several hours before sunrise.

Research in this area of study is partly justified by the comment of Garcia et al. (2010): “Frost damage is the leading weather hazard, on a planetary scale, as far as agricultural and forest economic losses are concerned”, and the work of Lee et al. (2014) on the short-term effect of temperature on daily emergency visits for acute myocardial infarction.

Nowcasting of the minima air, grass, or grass-surface temperature is essential if the impacts of frost, for example, are to be minimised and if active methods (Snyder and de Melo-Abreu 2005) such as the use of heaters, wind machines, or sprinkler irrigation for combatting frost are to be effective. Air temperature measurements are normally at heights of between 1.25 and 2 m, in a Stevenson screen or in a Gill radiation shield. Grass minimum temperature is measured using an unshielded thermometer, suspended just above short grass, according to the specifications provided by the World Meteorological Organisation (2008). An important indicator of frost occurrence at a remote/unattended site is the air, grass, or grass-surface temperatures. The rate of temperature reduction during the nighttime is influenced by, amongst other factors, wind speed, atmospheric water vapour pressure, atmospheric stability, precipitation, sky temperature and cloud type and amount (Savage 2012). During nighttime stable conditions, greatest temperature decreases occur under calm, dry and cloud-free conditions.

The models proposed for site-specific nowcasting of minimum air, grass and grass-surface temperatures are based on the assumption that 2–4 h is a sufficient notice period for active methods for combatting frost, such as sprinkler irrigation, heaters, fans and others, to be in place or for animals to be relocated to protected environments or for planning methods/protocols to be implemented by manufacturing enterprises and lay public. Various models have been used to determine the diurnal variation in temperature (air and soil) given measured daily maximum (*T*_x_) and minimum (*T*_n_) temperatures, day of year, time of day and site information (Groen 1947). For example, Johnson and Fitzpatrick (1977) proposed, for cloudless days and the absence of frontal weather systems, a method for estimating the diurnal temperatures during daylight hours based on measurements of *T*_x_ and *T*_n_. Based on *T*_x_ and *T*_n_, day of year/time of day, site information and three empirical constants, Parton and Logan (1981) used a sine-exponential model for estimating the diurnal variation in air and soil temperatures—sinusoidal from sunrise to sunset and exponential from sunset to the next sunrise. This model is physically plausible with the daytime sinusoidal component reproducing the temporal influence of solar irradiance and the exponential component reproducing Newton’s law of cooling for a heated surface after sunset and before sunrise the following day. Wann et al. (1985) compared a number of models and concluded that the sine-exponential model gave the best accuracy for estimating hourly temperatures using daily maxima and minima as data inputs. Snyder and de Melo-Abreu (2005) described the use of a square root model similar to that of Pelosi (1986) applied to hourly measurements—the Reuter algorithm—to predict the minimum air temperature using measurements from 2 h after sunset to sunrise the next morning.

Application of nighttime exponential and square root models, inverted in near real-time, were investigated so as to allow timely and accurate nowcasts of the minimum air temperature based on sub-hourly air temperature measurements and, therefore, the rate of temperature decrease, several hours before sunrise. The models were also applied to grass temperature and grass-surface temperature, the latter measured using an infrared thermometer at a weather station. The relative accuracy of the four models tested was investigated using historic temperature data (sub-hourly) at differing frequencies from selected sites at different altitudes. Furthermore, the implementation of a web-based system for display of the nowcasted temperatures in near real-time for one site is described.

In contrast to canopy climate models, such as SimSphere (Aberystwyth University 2014) for example, the method used depends on a strongly reduced set of input data. The data, composed exclusively of near real-time temperature measurements, makes the computation relocatable to the local scope (Russo and Coluccelli 2006).

## Theory

During stable nighttime conditions, in the absence of a frontal weather system and with cool, calm, cloud- and mist-free conditions, air temperature decreases continually reaching a minimum at around sunrise. For such conditions, Parton and Logan (1981) used a three-parameter model assuming that the air temperature *T*(*t*) (°C) at any time *t* (h) during the daytime can be determined from measurements of the daily maximum *T*_x_ (°C) and minimum *T*_n_ (°C) temperatures with nighttime temperatures determined using:

1 $$ T(t)={T}_{\mathrm{n}}+\left({T}_{\mathrm{ss}}-{T}_{\mathrm{n}}\right) \exp \left[\frac{-b\ \left(t-{t}_{\mathrm{ss}}\right)}{24-D}\right] $$

for time *t* before midnight where *T*_ss_ (°C) is the air temperature at sunset, denoted time *t*_ss_ (h), *b* = 2.2 is an empirically determined constant for air temperature measured about 1.5 m above ground, *D* (h) the day length for the site for the current day and 24 − *D* the night length. For times *t* after midnight, the period since sunset, *t* − *t*_ss_, is replaced by *t* + 24 − *t*_ss_. In Eq. (1), for the case of air temperature, the time lag between the minimum air temperature and that at sunrise has been ignored. Parton and Logan (1981) found this lag to be −0.17 h for air temperatures at a height of 1.5 m and −0.18 h at 0.1 m—that is, the minimum air temperatures occurred about 10 min before sunrise. Their sensitivity analysis showed that changes to this lag time resulted in only small increases in the air temperature estimation error. They further assumed that the daytime variation in air temperature is described by a truncated sine function:

2 $$ T(t)={T}_{\mathrm{n}}+\left({T}_{\mathrm{x}}-{T}_{\mathrm{n}}\right) \sin \left[\frac{\pi\ \left(t-{t}_{\mathrm{sr}}\right)}{D+2a}\right] $$

where *t*_sr_ is the sunrise time, and the time offset *a* (h), which has an approximate value of 1.86 h, is an empirically determined constant. The sunset temperature *T*_ss_ in Eq. (1) is estimated from *T*_n_, *T*_x_, *D* and *a* using Eq. (2):

3 $$ {T}_{\mathrm{ss}}={T}_{\mathrm{n}}+\left({T}_{\mathrm{x}}-{T}_{\mathrm{n}}\right) \sin \left(\frac{\pi\ D}{D+2a}\right). $$

For the square root model for nighttime air temperatures 2 h after sunset:

4 $$ T(t)=T\left({t}_{\mathrm{ss}+2}\right)-c\ \sqrt{t-{t}_{\mathrm{ss}+2}} $$

for *t* before midnight where *T*(*t*_ss + 2_) is the measured air temperature at *t*_ss + 2_, 2 h after sunset, *c* (°C h^-0.5^) is an empirically determined constant and *t* − *t*_ss + 2_ is the duration between *t* and 2 h after sunset. In Eqs. (1) and (4), for times after midnight, *t* − *t*_ss + 2_ is replaced by *t* + 24 − *t*_ss + 2_. By rearrangement of Eq. (4), the constant *c* for the period between *t*_ss + 2_ and *t*_sr_ may be determined from the (predicted) nowcasted air temperature minimum (*T*_pn_) and the measured air temperature *T*(*t*_ss + 2_):

5 $$ c=\left[{T}_{\mathrm{pn}}-T\left({t}_{\mathrm{ss}+2}\right)\right]/\sqrt{t_{\mathrm{sr}}+24-{t}_{\mathrm{ss}+2}} $$

where, for times *t*_ss + 2_ before midnight, *t*_sr_ + 24 − *t*_ss + 2_ is the period 2 h after sunset and before sunrise. The method is restricted to the period between 2 h after sunset *t*_ss + 2_ and sunrise *t*_sr_ since the net irradiance is relatively constant between these two times (Snyder and de Melo-Abreu 2005).

Four nowcasting models were tested for estimating the minimum air/grass/grass-surface temperature, 2 and 4 h before sunrise, based on sub-hourly temperature measurements between 4 and 2 h before sunrise for the 2-h nowcast and between 6 and 4 h before sunrise for the 4-h nowcast:

1. by inverting the exponential decay function (Eq. (1)) to solve for *T*_n_
2. by application of model 1 using *b* = 2.2
3. by application of the square root function based on temperature measurements 4 h before sunrise (Eq. 4)
4. by application of model 3 based on temperature measurements 2 h before sunrise.

These methods, applied in real-time either in a datalogger or in near real-time using a web-based system, may allow timely nowcasting of the minimum temperature based on sub-hourly temperature measurements. For this purpose, the nighttime exponential equation (Eq. (1)) was inverted and solved for *T*_n_ so as to nowcast the minimum temperature *T*_pn_:

6 $$ {T}_{\mathrm{pn}}=\left\{T(t)-{T}_{\mathrm{ss}} \exp \left[\frac{-b\ \left(t-{t}_{\mathrm{ss}}\right)}{24-D}\right]\right\}/\left\{1- \exp \left[\frac{-b\ \left(t-{t}_{\mathrm{ss}}\right)}{24-D}\right]\right\} $$

given, as an input, the measurement of temperature at time *t*, *T*(*t*), after midnight where:

7 $$ b=-\left(\frac{24-D}{t-{t}_{\mathrm{ss}}}\right) \ln \left[\frac{T(t)-{T}_{\mathrm{n}}}{T_{\mathrm{ss}}-{T}_{\mathrm{n}}}\right]. $$

For sub-hourly diurnal temperature data, which include the minimum air temperature, regressing ln [*T*(*t*) − *T*_n_] as a function of (*t* − *t*_ss_)/(24 − *D*) was assumed to yield a straight line with a slope of −*b*. For nowcasting, the empirical constant *b* may be determined, during several calm and cloud-free nights, several days preceding the calculation of *T*_pn_. Clouds and/or mist or rainfall and/or increased wind speed a few hours before sunrise could reverse or hinder the nighttime rate of air temperature decrease. A reversal of the expected temperature decrease for some of the time during the night could result in *b* < 0 and possibly an unreliable nowcast. Conversely, for the assumed *b* value, more rapid than expected temperature decreases could result in *T*_pn_ greater than *T*_n_. Changes in atmospheric conditions from one night to another could also result in different *b* values and therefore reduce the accuracy of the method.

In this study, the inversion of the exponential and the square root models, applied to the expected nighttime decrease in temperature (Eqs. (1) and (4)), is investigated. It is proposed that the methods be used to routinely nowcast the minimum temperature, given real-time measurement inputs of air/grass/grass-surface temperature 6 to 4 h (4-h nowcast) and 4 to 2 h (2-h nowcast) prior to the occurrence of the minimum temperature.

Generalising the nighttime exponential model (Eqs. (1) and (6)) to two measured temperatures *T*(*t*_1_) and *T*(*t*_2_) instead of *T*(*t*) and *T*_ss_, where *T*(*t*_2_) is a measured temperature at a later time *t*_2_ than temperature *T*(*t*_1_), with the two times several hours apart:

8 $$ {T}_{\mathrm{pn}}=\left\{T\left({t}_2\right)-T\left({t}_1\right) \exp \left[\frac{-b\ \left({t}_2-{t}_1\right)}{t_{\mathrm{sr}}-{t}_1}\right]\right\}/\left\{1- \exp \left[\frac{-b\ \left({t}_2-{t}_1\right)}{t_{\mathrm{sr}}-{t}_1}\right]\right\} $$

if times *t*_1_ and *t*_2_ are both before midnight or both after midnight. If *t*_1_ is before midnight and *t*_2_ after midnight, then

9 $$ {T}_{\mathrm{pn}}=\left\{T\left({t}_2\right)-T\left({t}_1\right) \exp \left[\frac{-b\ \left({t}_2+24-{t}_1\right)}{t_{\mathrm{sr}}+24-{t}_1}\right]\right\}/\left\{1- \exp \left[\frac{-b\ \left({t}_2+24-{t}_1\right)}{t_{\mathrm{sr}}+24-{t}_1}\right]\right\}. $$

The following four models are proposed for determining *T*_pn_, 2 or 4 h before sunrise, from pre-dawn sub-hourly temperature measurements.

- Model 1: inversion of exponential model using regression-determined *b*

This proposed method yields two model nowcasts using sub-hourly temperature measurement inputs between 6 and 4 h before sunrise for the first (4-h) nowcast and measurements 4 to 2 h before sunrise for the second (2-h) to determine the assumed exponential decrease in air temperature and hence to determine the exponential decay factor *b*. This factor together with the measured temperature inputs hours before sunrise are then used to obtain *T*_pn_ 4 and 2 h before sunrise. Modelled on the previous theory for a nighttime period (Eq. (8)) usually after midnight, for the 2-h-ahead nowcast, the sub-hourly temperature inputs *T*(*t*_sr − 4_) and *T*(*t*_sr − 2_) are used. At and between these times *t*, for which *t*_sr − 4_ ≤ *t* ≤ *t*_sr − 2_, the decay factor (*b* = *b*_sr − 4to−2_) was determined from the slope of the plot of ln [*T*(*t*) − min (*T*_sr − 4to−2_) + 0.01] vs $\frac{t-{t}_{\mathrm{sr}-4}}{t_{\mathrm{sr}-2}-{t}_{\mathrm{sr}-4}}$ where min (*T*_sr − 4to−2_) represents the minimum of the temperatures between the two times and *t*_sr − 2_ − *t*_sr − 4_ = 2 h. The constant of 0.01 °C ensures that the argument of the logarithm is always positive and hence defined when *T*(*t*) = min (*T*_sr − 4to−2_). Using this model, unlike the Parton and Logan (1981) method, a different *b* = *b*_sr − 4to−2_ value from *b* = 2.2 is determined for each early-morning period. Similar procedures were used for the 4-h ahead nowcasts using temperatures between times *t*_sr − 6_ and *t*_sr − 4_ and the decay factor *b* = *b*_sr − 6to−4_ from the slope of the plot of ln [*T*(*t*) − min (*T*_sr − 6to− 4_) + 0.01] vs $\frac{t-{t}_{\mathrm{sr}-6}}{t_{\mathrm{sr}-4}-{t}_{\mathrm{sr}-6}}$.

By inversion of Eq. 1 (Eqs. (8) or (9)), and application to the period 4 to 2 h before sunrise for a nowcast 2 h before sunrise, *T*_pn_ was determined assuming a continued and exponential decay after *t*_sr − 2_, at the same exponential rate:

10 $$ {T}_{\mathrm{pn}} = \left\{T\left({t}_{\mathrm{sr}-2}\right)-T\left({t}_{\mathrm{sr}-4}\right) \exp \left[\frac{-{b}_{\mathrm{sr}-4\mathrm{t}\mathrm{o}-2}\ \left({t}_{\mathrm{sr}-2}-{t}_{\mathrm{sr}-4}\right)}{t_{\mathrm{sr}}-{t}_{\mathrm{sr}-4}}\right]\right\}/\left\{1- \exp \left[\frac{-{b}_{\mathrm{sr}-4\mathrm{t}\mathrm{o}-2}\ \left({t}_{\mathrm{sr}-2}-{t}_{\mathrm{sr}-4}\right)}{t_{\mathrm{sr}}-{t}_{\mathrm{sr}-4}}\right]\right\} $$

which for this 2-h nowcast simplifies to:

11 $$ {T}_{\mathrm{pn}}=\left[T\left({t}_{\mathrm{sr}-2}\right)-T\left({t}_{\mathrm{sr}-4}\right) \exp\ \left(-{b}_{\mathrm{sr}-4\mathrm{t}\mathrm{o}-2}/2\right)\right]/\left[1- \exp\ \left(-{b}_{\mathrm{sr}-4\mathrm{t}\mathrm{o}-2}/2\right)\right]. $$

For a nowcast 4 h before sunrise:

12 $$ {T}_{\mathrm{pn}} = \left[T\left({t}_{\mathrm{sr}-4}\right)-T\left({t}_{\mathrm{sr}-6}\right) \exp\ \left(-{b}_{\mathrm{sr}-6\mathrm{t}\mathrm{o}-4}/3\right)\right]/\left[1- \exp\ \left(-{b}_{\mathrm{sr}-6\mathrm{t}\mathrm{o}-4}/3\right)\right]. $$

The equations and methods used for historic data or for an on-board datalogger 2-h before sunrise nowcast are shown in Table 1 and similarly using Eq. (12) applied for the minimum between 6 and 4 h before sunrise for the 4-h before sunrise nowcast. For the 2-h nowcast for times when the calculated *b* value was out of its expected range, typically |*b*| < 1, then Eq. (11) was applied using *b*_sr − 4to−2_ = 2.2 and *T*(*t*_sr − 2_) replaced by the minimum temperature between 4 and 2 h before sunrise. A similar procedure was followed for the 4-h nowcast using Eq. (12) and *b*_sr − 6to−4_ = 2.2.

**Table 1:** Equations and datalogger methods used for the 2-h nowcasting of the daily minimum temperature

Model	Determination/value of constant^a^	Calculation^b^ of *T*_pn_	Conditions for trapping rare nighttime increases in temperature
1	*b* determined from the slope of the plot of ln [*T*(*t*) − min (*T*_sr − 4 to − 2_) + 0.01] vs (*t* − *t*_sr − 4_)/(*t*_sr − 2_ − *t*_sr − 4_): *b*_sr − 4to− 2_ = − [covariance ($\frac{t-{t}_{\mathrm{sr}-4}}{t_{\mathrm{sr}-4}-{t}_{\mathrm{sr}-2}}, \ln\ \Big(T(t)- \min\ \left({T}_{\mathrm{sr}-4\mathrm{t}\mathrm{o}-2}\right)+0.01$)]/variance_p_ $\left(\frac{t-{t}_{\mathrm{sr}-4}}{t_{\mathrm{sr}-2}-{t}_{\mathrm{sr}-4}}\right)$	${T}_{\mathrm{pn}}=\frac{T\left({t}_{\mathrm{sr}-2}\right)-T\left({t}_{\mathrm{sr}-4}\right) \exp\ \left(-{b}_{\mathrm{sr}-4\mathrm{t}\mathrm{o}-2}/2\right)}{1- \exp \left(-{b}_{\mathrm{sr}-4\mathrm{t}\mathrm{o}-2}/2\right)}$	If (|*b*_sr − 4 to − 2_| < 1 then ${T}_{\mathrm{pn}}=\left[ \min\ \left({T}_{\mathrm{sr}-4\mathrm{t}\mathrm{o}-2}\right)-{T}_{\mathrm{sr}-4}\ \exp \left(-\frac{2.2}{2}\right)\right]/\left[1- \exp \left(-\frac{2.2}{2}\right)\right]$ else if (*b*_sr − 4 to − 2_ < 0 or *T*_sr − 4_ < *T*_sr − 2_) then *T*_pn_ = min (*T*_sr − 4 to − 2_) else ${T}_{\mathrm{pn}}=\left[{T}_{\mathrm{sr}-2}-{T}_{\mathrm{sr}-4}\ \exp \left(-\frac{b_{\mathrm{sr}-4\mathrm{t}\mathrm{o}-2}}{2}\right)\right]/\left[1- \exp \left(-\frac{b_{\mathrm{sr}-4\mathrm{t}\mathrm{o}-2}}{2}\right)\right]$
2	*b* = 2.2	${T}_{\mathrm{pn}}=\frac{T\left({t}_{\mathrm{sr}-2}\right)-T\left({t}_{\mathrm{sr}-4}\right) \exp\ \left(-2.2/2\right)}{1- \exp\ \left(-2.2/2\right)}$	If (|*b*_sr − 4 to − 2_| < 1 then ${T}_{\mathrm{pn}}=\left[ \min\ \left({T}_{\mathrm{sr}-4\mathrm{t}\mathrm{o}-2}\right)-{T}_{\mathrm{sr}-4}\ \exp \left(-\frac{2.2}{2}\right)\right]/\left[1- \exp \left(-\frac{2.2}{2}\right)\right]$ else if (*b*_sr − 4 to − 2_ < 0 or *T*_sr − 4_ < *T*_sr − 2_) then *T*_pn_ = min (*T*_sr − 4 to − 2_) else ${T}_{\mathrm{pn}}=\left[{T}_{\mathrm{sr}-2}-{T}_{\mathrm{sr}-4}\ \exp \left(-\frac{2.2}{2}\right)\right]/\left[1- \exp \left(-\frac{2.2}{2}\right)\right]$
3	*c* is determined from the slope of a plot of *T*(*t*) − *T*(*t*_sr − 4_) vs $\sqrt{t-{t}_{\mathrm{sr}-4}}$: *c*_sr − 4 to − 2_ = − [covariance (*T* − *T*_sr − 4_, $\sqrt{t-{t}_{\mathrm{sr}-4}}$]/variance_p_ ($\sqrt{t-{t}_{\mathrm{sr}-4}}$) for temperatures 4 to 2 h before sunrise	*T*_pn_ = *T*_sr − 4_ − 2 *c*_sr − 4 to − 2_	If *c*_sr − 4 to − 2_ < 0 or *T*_sr − 4_ < *T*_sr − 2_ then *T*_pn_ = min (*T*_sr − 2 to − 4_) else *T*_pn_ = *T*_sr − 4_ − 2 *c*_sr − 4 to − 2_
4	*c*_sr − 4 to − 2_ = − [covariance (*T* − *T*_sr − 4_, $\sqrt{t-{t}_{\mathrm{sr}-4}}$]/variance_p_ ($\sqrt{t-{t}_{\mathrm{sr}-4}}$) for temperatures 4 to 2 h before sunrise	${T}_{\mathrm{pn}}={T}_{\mathrm{sr}-2}-\sqrt{2}\ {c}_{\mathrm{sr}-4\mathrm{t}\mathrm{o}-2}$	If (*c*_sr − 4 to − 2_ < 0 or *T*_sr − 4_ < *T*_sr − 2_) then *T*_pn_ = min (*T*_sr − 4 to − 2_) else ${T}_{\mathrm{pn}}={T}_{\mathrm{sr}-2}-\sqrt{2}{c}_{\mathrm{sr}-4\mathrm{t}\mathrm{o}-2}$

^a^Covariance, variance and slope, where for example, *b* = − slope = − covariance (*X*, *Y*)/variance_p_ (*X*), performed 2 h before sunrise based on 2-min temperature measurements 4 to 2 h before sunrise where variance_p_ is the population variance

^b^Calculations performed 2 h before sunrise based on temperature measurements 4 to 2 h before sunrise

- Model 2 application of exponential model with *b* = 2.2

This proposed model, instead of the value for *b* (=*b*_sr − 4 to − 2_ for the 2-h nowcast and *b* = *b*_sr − 6 to− 4_ for the 4-h nowcast) determined by regression (Table 1), uses a fixed value of 2.2 (Parton and Logan 1981) in Eqs. (11) and (12), respectively. In the case of real-time analyses, this model is simple since no on-board datalogger real-time regression analysis is required. Model 2 is also used as part of model 1 when |*b*| < 1.

- Model 3: application of square root model using *T*_sr − 4_

For this model, *T*_pn_ is determined for times 4 and 2 h before sunrise based on the square root model (Eq. 4) using the relationship:

13 $$ T(t)=T\left({t}_{t_{\mathrm{sr}-4}}\right)-{c}_{\mathrm{sr}-4\mathrm{t}\mathrm{o}-2}\ \sqrt{t-{t}_{\mathrm{sr}-4}}. $$

Therefore, using temperature measurements between 4 and 2 h before sunrise, a plot of *T*(*t*) − *T*(*t*_sr − 4_) vs $\sqrt{t-{t}_{\mathrm{sr}-4}}$ yields a slope of − *c*_sr − 4 to − 2_ from which *T*_pn_ is determined using:

14 $$ {T}_{\mathrm{pn}}=T\left({t}_{\mathrm{sr}-4}\right)-{c}_{\mathrm{sr}-4\mathrm{t}\mathrm{o}-2}\ \sqrt{t_{\mathrm{sr}}-{t}_{\mathrm{sr}-4}} $$

which simplifies to

15 $$ {T}_{\mathrm{pn}}=T\left({t}_{\mathrm{sr}-4}\right)-2\ {c}_{\mathrm{sr}-4\mathrm{t}\mathrm{o}-2} $$

where it is assumed that the same *c*_sr − 4 to −2_ can also be used for times 2 h before sunrise and sunrise. A modified version of Eq. (15) was used for the 4-h before sunrise nowcasts based on *c*_sr − 6 to − 4_ and temperature measurements between times 6 and 4 h before sunrise.

- Model 4: application of modified square root model, using *T*_sr − 2_

This model is the same as model 3 for determining *T*_pn_ for times *t* between 4 and 2 h before sunrise but with *T*(*t*_sr − 2_) replacing *T*(*t*_sr − 4_):

16 $$ {T}_{\mathrm{pn}}=T\left({t}_{\mathrm{sr}-2}\right)-{c}_{\mathrm{sr}-4\mathrm{t}\mathrm{o}-2}\ \sqrt{t_{\mathrm{sr}}-{t}_{\mathrm{sr}-2}}. $$

This simplifies to

17 $$ {T}_{\mathrm{pn}}=T\left({t}_{\mathrm{sr}-2}\right)-\sqrt{2}\ {c}_{\mathrm{sr}-4\mathrm{t}\mathrm{o}-2} $$

where it is assumed that the same *c*_sr − 4 to −2_ can be used for times between 2 h before sunrise and sunrise. As was the case for model 3 (square root), a modified version of Eq. (17) was used for the 4-h before sunrise nowcasts.

The various equations and datalogger protocols used in the datalogger for the web-based early-warning system used are outlined in Table 1. These protocols were also used for the spreadsheet calculations for the historic air temperature data for all sites.

All model nowcasts, a few hours before sunrise, are compromised by events such as transient clouds, increased wind speed, changes in atmospheric stability and precipitation with consequential likely disagreement between model nowcasts and measurements. Usually, in the case of frost, however, these events tend to reduce the chance of freezing conditions.

## Materials and methods

This work involves an agrometeorological application of a web-based data and information system (Savage 2014; Savage et al. 2014) and the nowcasting of daily minima temperatures, for frost prediction for example, using various models for a short-grass surface with the results displayed and updated automatically in near real time. Methods were applied to near real-time temperature measurements from Pietermaritzburg (South Africa) and to historic data from three other subtropical sites and data from one site in central sub-Saharan Africa (Table 2, column 1). Sub-hourly temperature data, predominantly air temperature from the five sites varying in altitude from 30 to nearly 2000 m, were used. The relevant details of the various weather station systems, sensors, datalogging and telecommunication equipment and data used are shown in Table 2 (columns 2 and 3). For Pietermaritzburg, air temperature and relative humidity at 2 m were measured in a naturally ventilated six-plate Gill radiation shield. For this site, which included grass-minimum and grass-surface temperatures, data for 2011 and 2012 were used. The data for part of 2011 were used for model development with 2012 data used for testing goodness of model fit. Grass temperature for this site was measured in accordance with the World Meteorological Organization (2008) guidelines for sensor exposure. A 25-mm length of chromel-constantan thermocouple wire was freely exposed 25–50 mm above the soil surface so as to be in contact with blades of grass. Grass-canopy surface temperature was measured using an 8–14-μm germanium lens infrared thermometer (IRT) positioned at 45° to the horizontal, facing south and at a height of 2.5 m, sensing a grass target diameter of 1.9 m. The thermocouple for grass temperature was calibrated, in a water bath, against a reference PT1000 resistance thermometer (data not shown) and the IRT calibrated using a large radiator (Savage and Heilman 2009). No corrections for the thermocouple temperatures were applied. Corrections were applied for surface temperatures based on the IRT voltage output and the sensor body temperature. For these grass-surface temperatures at a weather station, the grass fully covered the soil. All temperature measurements were performed differentially every 15 s and averaged every 2 min. This allowed a sufficiently large sample number (60) of data pairs for the linear regression statistics for the 2- and 4-h before sunrise nowcasts (Table 1).

**Table 2:** Location, datalogging, sensor and data details

Station details	Field-station sensor details	Data
Pietermaritzburg, mast 1, South Africa (altitude, 684 m; latitude, 29.628° S; longitude, 30.403° E)	CR1000^a^ datalogger and AM32A^a^ multiplexer. IRT^b^ at 2.6 m; unshielded chromel-constantan thermocouple (24–gauge) for grass temperature at 25 to 50 mm above soil surface; CS500^a^ in 6-plate Gill shield Datalogger-attached RF416^a^ broad-spectrum radio, panel antenna in line-of-sight with base station Field station antenna connected to an arrestor, in turn connected to radio. The datalogger was earthed. A RF416 radio connected to an 8-m antennae and surge-protector. Base station software included LoggerNet^a^ for data downloads	2-min surface, grass and air temperature measurements, the latter at 2 m, for 21st April to 18th August 2011
Marianna, Tower 130, Jackson County, FL, USA (altitude, 35 m; latitude, 30.850° N; longitude, 85.165° W)	CS107^a^ air temperature sensor in 12-plate Gill shield at 0.6-m height; CS215^a^ air temperature and RH instrument in 12-plate Gill shield at 2 m CR10X^a^ datalogger with attached RF401^a^ radio and cell modem	15-min air temperature measurements for 2004 for 0.6- and 2-m heights
Cedara, South Africa (altitude, 1076 m; latitude, 29.5333° S; longitude, 30.2833° E)	TR1^c^ air temperature and relative humidity sensor ETo^a^ datalogger station	15-min air temperature measurements for 1st January 2005 to 17th April 2006 for 2-m height
Cathedral Peak, South Africa (altitude, 1935 m; latitude, 29.4833° S; longitude, 30.5° E)	Unshielded 75-μm chromel-constantan thermocouples and 21X^a^ datalogger	20-min air temperature measurements for 1992 for 0.5- and 1.5-m heights
Kinsevere, DRC, central sub-Saharan Africa (altitude, 1243 m; latitude, 11.36433° S; longitude, 27.5646° E)	CR1000^a^ datalogger with HMP50^a^ in 6-plate Gill shield	1-min air temperature measurements for 2014 (10 Jan to 19 Aug inclusive) for 2-m height

^a^Campbell Scientific Inc., Logan, UT, USA

^b^Apogee IRT model IRR-P (half angle of 22°): Apogee Instruments Inc., Logan, UT, USA

^c^Adcon Telemetry GmbH, Inkustrassse 24, A-3400 Klosterneuburg, Austria

The calculations for daylength and sunrise time, required for all four models, were included in the datalogger program using VBA functions provided by the National Oceanic and Atmospheric Administration: http://www.srrb.noaa.gov/highlights/sunrise/calcdetails.html

The applicability of both the nighttime exponential and square root models was investigated, the former model inverted so as to nowcast the minimum temperature from inputs of sub-hourly temperature measurements several hours before sunrise.

Use of hourly temperature data, for which only two temperatures would be available 2 h before sunrise, would not allow the necessary slope calculations using the exponential and square root models and would result in a two-point model. Hence, more frequent data collection was used. For the nowcasts for the Pietermaritzburg site, once the temperatures for the period 4 to 2 h before sunrise had been stored, they were recalled from datalogger memory, reformulated as necessary for the exponential and square root models for linearisation of the model relationships and then a covariance instruction, part of the datalogger instruction set, applied to obtain the slope values for the 2-h nowcasts (*b*_sr − 4 to − 2_ and *c*_sr − 4 to − 2_ for the respective models) from covariance and population variance instructions (Table 1). These instructions allowed *T*_pn_ to be determined 2 and 4 h before sunrise.

All historic datasets used contained sub-hourly air temperatures (Table 2). The four models (Table 1) were applied using 15-min air temperature measurements for 2004 for 0.6- and 2-m heights for Marianna, Jackson County, FL, USA (Table 2). For this dataset (ftp://if-fwn-prdw01.osg.ufl.edu/fawnpub/data/15_minute_obs/), model nowcasts were made 2 h before sunrise using air temperature measurements 4 to 2 h before sunrise as well as nowcasts 4 h before sunrise using measurements 6 to 4 h before sunrise. For Cedara and Cathedral Peak, Catchment VI (South Africa), 15- and 20-min air temperature data were used, respectively. In the case of Cathedral Peak, air temperature measurements at 0.5- and 1.5-m heights were used (Table 2). All datasets were from sites in the subtropics except for data from Kinsevere in central sub-Saharan Africa (Katanga province of the Democratic Republic of the Congo at almost 11° S) (Table 2).

In the case of model 1, in an attempt to trap events for which the nighttime temperature increases due to clouds or other meteorological influences, conditions were imposed based on a calculated slope value *b* or that the temperature at *t*_sr − 2_ exceeds that at *t*_sr − 4_. For this model, nowcasts with a |*b*| value <1 resulted in large deviations between *T*_pn_ and *T*_n_. For |*b*| < 1, *T*_pn_ for the 2-h nowcast was estimated using Eq. (11) using *b* = 2.2 and *T*(*t*_sr − 2_) = min (*T*_sr − 4to−2_). The latter ensured that the lowest measured temperature for this time period was used in the computation. If temperatures were increasing during the night with the result that *T*(*t*_sr − 2_) > *T*(*t*_sr − 4_) or the computed *b* was negative, *T*_pn_ was assigned the minimum temperature between the two times, or else Eq. (11) was applied using the computed *b*_sr − 4 to − 2_. The conditions for trapping data for which there were nighttime increases in temperature for model 2 for which *b* = 2.2 and for the 4-h nowcasts (Eq. (12)) were similar to those for model 1 (Table 1, column 3).

In the case of Pietermaritzburg, for nowcasting the minimum temperatures (air, grass and grass-surface), email alerts were used and near real-time data displayed, *T*_pn_ in particular, on the Internet using an open web-based data and information system described by Savage (2014) and Savage et al. (2014).

Comparisons between the four model determinations of the nowcasted daily minimum air temperature *T*_pn_ for the five sites were compared against the actual daily air temperature minimum *T*_n_ using statistical analyses and regression scatter plots. Confidence intervals (99 and 95 %) for slopes and intercepts were determined to test for significant differences from 1 to 0 °C respectively.

## Results and discussion

### Pietermaritzburg 2-min measurements of air, grass and grass-surface temperature

**Fig. 1:**
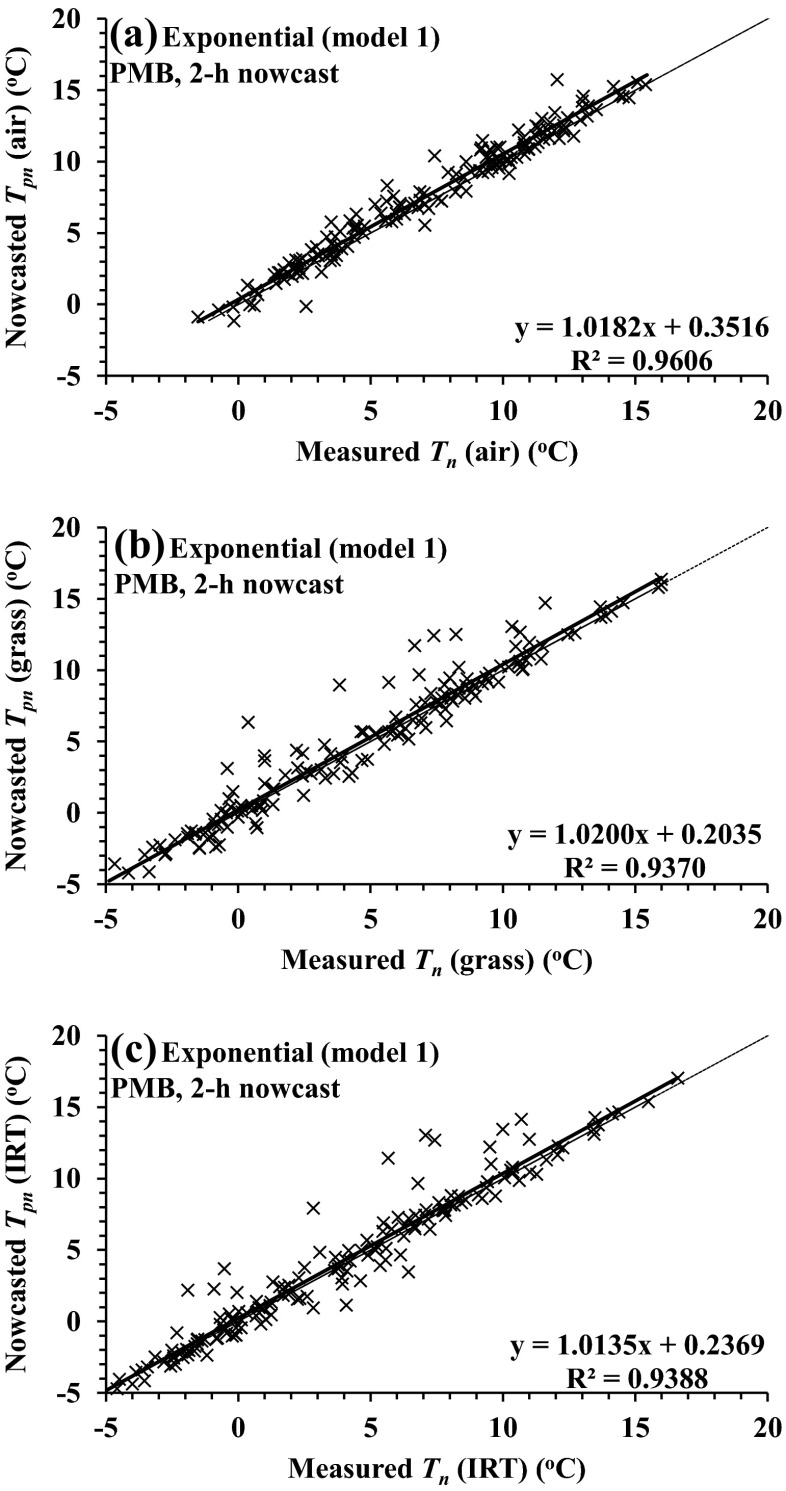
Regression plots for the experimental exponential model 1 for the 2-h-ahead nowcasted for Pietermaritzburg data: **a** for model-nowcasted minimum air temperature (*T*_pn_) vs measured minimum air temperature (*T*_n_), **b** for model-nowcasted *T*_pn_ (grass) vs *T*_n_ (grass) and **c** for model-nowcasted *T*_pn_ (IRT) vs *T*_n_ (IRT). The regression line (*thick black*) and 1:1 lines (*thinner grey*) are shown

**Fig. 2:**
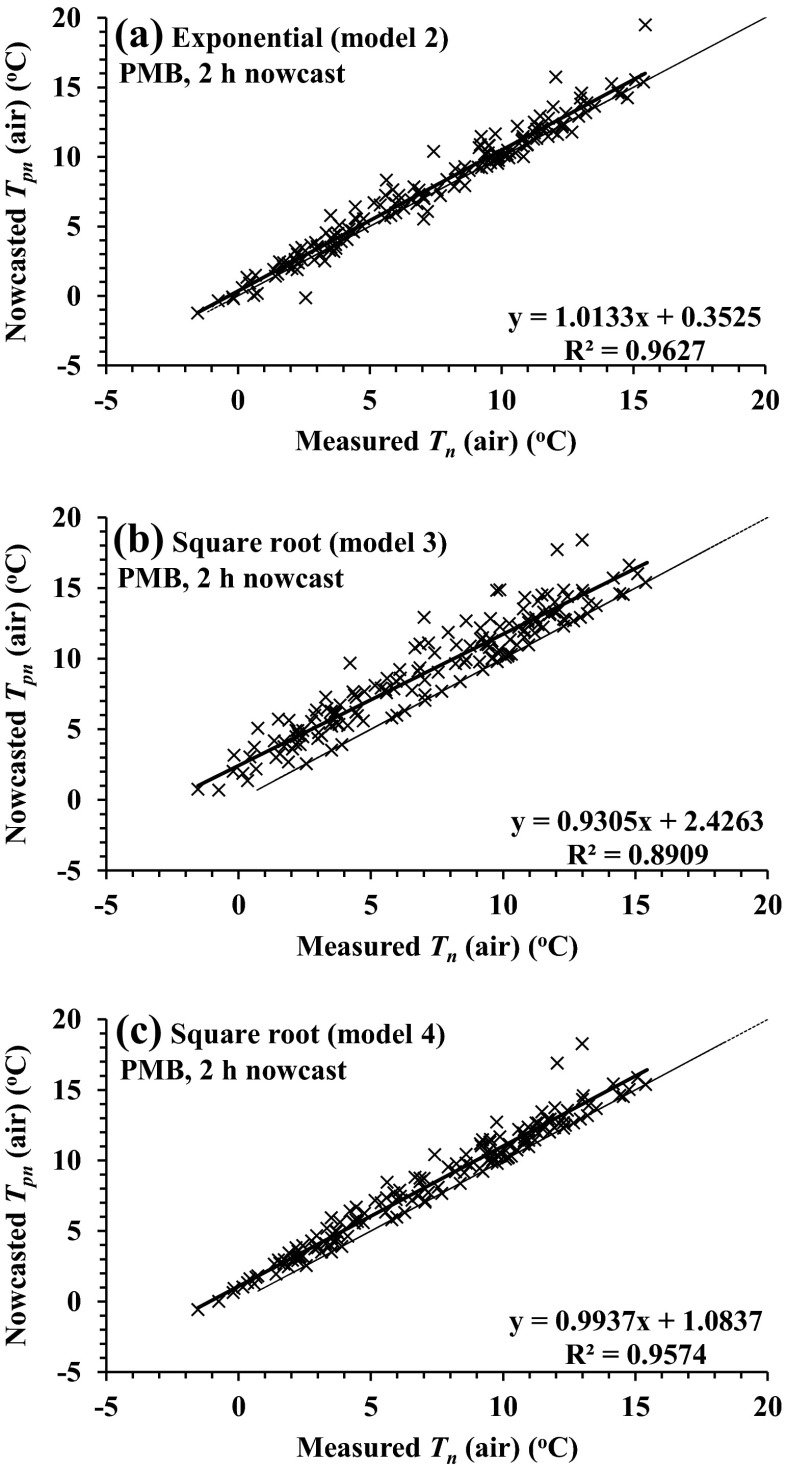
Regression plots for the 2-h-ahead nowcasts for Pietermaritzburg air temperature (*T*_pn_) vs measured minimum air temperature (*T*_n_) for **a** exponential model 2 nowcasts, **b** square root model 3 (using *T*(*t*_sr − 4_)) and **c** square root model 4 (using *T*(*t*_sr − 2_))

Judging by the increased root mean square error (RMSE), the *T*_pn_ (nowcasted) vs *T*_n_ (measured) 2-m air temperature comparisons for the nowcasts 2 h before sunrise were less variable than for the grass and grass-surface (IRT) temperatures (Table 3, see Pietermaritzburg, 2011; Fig. 1a compared to b and c). Model 1 (exponential, Eq. (11)) 2-h nowcast comparisons (*T*_pn_) against measurements (*T*_n_) yielded RMSE values of 0.870, 1.349 and 1.329 °C for air, grass and grass-surface temperatures, respectively, and a mean bias error (MBE) of <0.5 °C (Table 3, see Pietermaritzburg, 2011). For the 2- and 4-h nowcasts before sunrise, the differences between the air, grass and grass-surface temperature regression slopes and intercepts from 1 to 0 °C respectively for models 1 and 2 were small (Tables 3 (see Pietermaritzburg, 2011) and 4 (see Pietermaritzburg, 2011)). Model 3 and 4 comparisons were generally more variable than those for models 1 and 2 (Fig. 2b and c compared to Figs. 1a and 2a) and usually characterised by decreased coefficient of determination (*R*^2^) and increased RMSE and MBE. Furthermore, models 3 and 4 comparisons against *T*_n_ measurements were usually characterised by a larger (positive) intercept compared to those for models 1 and 2 (Table 3, see Pietermaritzburg, 2011; Figs. 1a and 2).

**Table 3:** Model statistics for comparisons of *T*_n_ with *T*_pn_, the latter 2 h before sunrise: Pietermaritzburg 2-min air (2 m), grass (25–50 mm) and surface temperature minimum temperature determinations for the 2011 data set, (the most accurate determinations—usually for model 1—are in italics), Marianna 15-min air (0.6 and 2 m) temperature minimum temperature nowcasts for the 2004 data set, Cedara 15-min air (2 m) temperature for the 2005–2006 data set (1st January 2005 to 17th April 2006), Cathedral Peak 20-min air (0.5 and 1.5 m) temperature for the 1992 data set and Kinsevere, DRC 1-min air temperature (2 m) for the 2014 data set

Measurement	Model	Slope	Intercept (°C)	*R* ^2^	RMSE (°C)	MBE (°C)	*n* ^†^	*b* or *c* ^††^	*f* ^†††^
Pietermaritzburg, 2011									
Air temperature (2 m)	1	1.018	0.352d	0.961	0.870	0.484	158	2.131	26
	*2*	*1.013*	*0.353cd*	*0.963*	*0.841*	*0.369*	*158*	*2.2*	
	3	0.930ab	2.426cd	0.891	1.374	1.920	159	0.908	23
	4	0.994	1.084cd	0.957	0.884	1.080	159	0.908	
Grass temperature (25–50 mm)	*1*	*1.020*	*0.203*	*0.937*	*1.349*	*0.297*	*160*	*1.470*	*29*
	2	0.998	0.440cd	0.939	1.298	0.438	160	2.2	
	3	0.934b	2.584cd	0.870	1.851	2.280	158	1.208	27
	4	0.979	1.336cd	0.940	1.268	1.324	158	1.208	
Grass-surface temperature	*1*	*1.014*	*0.237*	*0.939*	*1.329*	*0.289*	*158*	*1.600*	*26*
	2	1.009	0.283d	0.932	1.398	0.291	158	2.2	
	3	0.990	2.150cd	0.859	2.056	2.111	159	1.160	29
	4	1.005	1.090cd	0.937	1.337	1.093	159	1.160	
Marianna, Jackson County, 2004									
Air temperature (0.6 m)	*1*	*0.991b*	*0.344cd*	*0.994*	*0.621*	*0.222*	*351*	*2.591*	*21*
	2	0.995	0.174d	0.993	0.650	0.164	361	2.2	
	3	1.000	−0.161	0.990	0.785	−0.158	351	0.395	19
	4	0.996	0.074	0.992	0.691	0.074	360	0.395	
Air temperature (2 m)	*1*	*0.992*	*0.367cd*	*0.994*	*0.620*	*0.252*	*361*	*2.681*	*19*
	2	0.996	0.167d	0.993	0.643	0.160	361	2.2	
	3	1.003	−0.173d	0.990	0.773	−0.156	360	0.401	22
	4	0.997	0.066	0.992	0.686	0.066	360	0.401	
Cedara, 2005–2006									
Air temperature (2 m)	*1*	*0.988*	*0.529cd*	*0.973*	*0.710*	*0.388*	*465*	*2.172*	*38*
	2	0.989	0.441cd	0.970	0.753	0.432	465	2.2	
	3	0.989	0.317cd	0.961	0.857	0.190	464	0.432	34
	4	0.987	0.501cd	0.969	0.758	0.499	464	0.432	
Cathedral Peak, 2002									
Air temperature (0.5 m)	*1*	*1.008*	*0.293cd*	*0.960*	*0.957*	*0.358*	*351*	*2.318*	*35*
	2	1.019	0.107	0.959	0.987	0.038	358	2.2	
	3	1.014	−0.022	0.938	1.217	0.163	350	0.539	34
	4	1.018	0.071	0.962	0.950	0.060	357	0.539	
Air temperature (1.5 m)	*1*	*0.998*	*0.388cd*	*0.964*	*0.910*	*0.373*	*358*	*2.316*	*33*
	2	1.010	0.191	0.962	0.950	−0.111	358	2.2	
	3	1.012	0.014	0.941	1.198	0.115	357	0.526	34
	4	1.005	0.273cd	0.954	1.038	0.273	357	0.526	
Kinsevere, Katanga province, DRC 2014									
Air temperature (2 m)	*1*	*0.966b*	*0.600cd*	*0.956*	*0.825*	*0.126*	*188*	*1.792*	*14*
	2	0.971b	0.571cd	0.962	0.770	0.167	188	2.2	
	3	1.001	−0.134d	0.929	1.098	−0.123	188	0.594	11
	4	0.960b	0.730	0.950	0.883	0.724	188	0.594	

Model 1: application of exponential model; model 2: inversion of exponential model to sunrise; model 3: application of square root model. a and/or b denotes significant difference from a slope of 1 at 99 and 95 % levels respectively; c and/or d denotes significant difference from an intercept of 0 °C at 99 and 95 % levels respectively

^†^ *n* is the number of data pairs. Days for the *T*_n_, *T*_pn_ regression comparisons

^††^ *b* (exponential model) or *c* (square root model) refers to an average value. Alternatively, a fixed value of *b* = 2.2 was used

^†††^ *f* is the percentage of values replaced using the conditional statements of Table 1

**Table 4:** Model statistics for 4-h nowcasts: Pietermaritzburg, Marianna, Cedara, Cathedral Peak and Kinsevere, DRC. The most accurate model determinations are italicised. Model 1 was usually most suitable in all cases, except for Kinsevere

Measurement	Model	Slope	Intercept (°C)	*R* ^2^	RMSE (°C)	MBE (°C)	*n*	*b* or *c*	*f*
Pietermaritzburg, 2011									
Air temperature (2 m)	1	1.014	0.630cd	0.891	1.466	0.729	165	1.908	24
	*2*	*1.062*	*0.701cd*	*0.890*	*1.463*	*0.708*	*165*	*2.2*	
	3	1.015	0.152	0.867	1.653	0.261	157	1.024	22
	4	0.978	0.880cd	0.896	1.391	0.866	157	1.024	
Grass temperature (25–50 mm)	*1*	*1.014*	*0.311cd*	*0.882*	*1.861*	*0.374*	*163*	*1.491*	*26*
	2	0.902	0.448cd	0.862	1.807	0.365	163	2.2	
	3	1.012	0.014	0.709	3.214	0.067	159	1.443	24
	4	0.998	0.632d	0.800	2.477	0.630	159	1.443	
Grass-surface temperature	*1*	*1.038*	*0.168cd*	*0.861*	*1.970*	*0.258*	*145*	*1.610*	*29*
	2	1.033	0.205cd	0.837	2.158	0.234	145	2.2	
	3	1.058	−0.315	0.756	3.045	−0.101	157	1.348	26
	4	1.042	0.342	0.831	2.381	0.373	157	1.348	
Marianna, Jackson County, 2004									
Air temperature (0. 6 m)	1	0.991	0.595cd	0.989	0.838	0.474	351	2.723	18
	*2*	*1.007*	*0.047*	*0.989*	*0.881*	*0.061*	*360*	*2.2*	
	3	1.004	0.151	0.988	0.883	0.209	352	0.490	17
	4	1.006	−0.181	0.985	0.972	−0.179	352	0.490	
Air temperature (2 m)	1	0.991	0.639cd	0.988	0.842	0.543	361	2.391	17
	2	1.005	0.088cd	0.988	0.878	0.091	361	2.2	
	*3*	*1.004*	*0.132*	*0.989*	*0.796*	*0.196*	*352*	*0.509*	*17*
	4	0.996	0.510cd	0.989	0.819	0.509	352	0.509	
Cedara, 2005–2006									
Air temperature (2 m)	1	1.007	0.503cd	0.945	1.036	0.583	471	2.185	33
	2	1.020	0.217cd	0.936	1.140	0.239	471	2.2	
	3	1.018	0.402cd	0.937	1.130	0.608	464	3	20
	*4*	*1.006*	*0.711cd*	*0.944*	*1.048*	*0.712*	*464*	*4*	
Cathedral Peak, 2002									
Air temperature (0.5 m)	*1*	*0.993*	*0.522cd*	*0.926*	*1.308*	*0.472*	*351*	*2.396*	*29*
	2	1.008	0.104cd	0.884	1.683	0.112	362	2.2	
	3	0.998	0.394d	0.887	1.657	0.378	362	0.627	30
	4	0.988	0.719cd	0.911	1.433	0.716	362	0.627	
Air temperature (1.5 m)	*1*	*0.989*	*0.504cd*	*0.927*	*1.297*	*0.417*	*362*	*2.347*	*29*
	2	1.053	0.497cd	0.852	2.057	0.555	362	2.2	
	3	0.993	0.428d	0.894	1.600	0.370	362	0.603	30
	4	0.983	0.748cd	0.917	1.388	0.774	362	0.603	
Kinsevere, Katanga province, DRC 2013–2014									
Air temperature (2 m)	1	0.813ab	3.651cd	0.881	1.182	1.053	188	2.182	18
	2	0.842ab	3.106cd	0.879	1.239	0.913	188	2.2	
	*3*	*0.852ab*	*2.967cd*	*0.861*	*1.354*	*0.328*	*188*	*0.670*	*20*
	4	0.811ab	3.837cd	0.890	1.129	3.630	188	0.670

For model 1 (exponential) for the 2-h nowcasts, the average *b* value for air temperature (*b* = 2.131) was <2.2 and even less for the grass (1.470) and grass-surface (1.600) temperatures (Table 3). Only positive *b* values were included in the averaging. A decreased *b* value implies a reduced rate of reduction in the nighttime temperatures. For the 159-day period, about 25 % of all nights contained negative *b* values or nights for which *T*(*t*_sr − 2_) > *T*(*t*_sr − 4_) (Table 3, last column).

In general, models 1 and 2 (exponential) and 4 (square root) performed well in determining *T*_*pn*_ for the 2-h nowcast with model 1 usually the best. For the 4-h air, grass, grass-surface nowcasts, *R*^2^ decreased, particularly for the grass and grass-surface temperatures, and RMSE increased by between about 15 and 100 % for the different models (Table 3 (Pietermaritzburg, 2011) cf. 4 (Pietermaritzburg, 2011)). As was the case for the 2-h nowcasts, use of model 3 (square root) yielded less accurate 4-h nowcasts with increased *R*^2^ and increased RMSEs.

### Measurements of air temperature for all other sites

For both the 2- and 4-h nowcasts of air temperature, exponential model 1 used the *b* coefficient determined 2- or 4-h before sunrise to determine *T*_pn_. The 15- or 20-min *T*_pn_ (nowcasted) vs *T*_n_ (measured) air temperature slopes for the 2-h nowcasts for all heights and models were most often not statistically different from a slope of 1. As was the case for the Pietermaritzburg comparisons, model 1 (exponential) was marginally better than the other models for all sites. Application of models 1 and 2 (exponential), for Cathedral Peak, a high-altitude site with frequent mists in summer, resulted in the largest RMSE for both the 0.5- and 1.5-m measurement heights. Model 3 (square root) 2-h nowcasts resulted in the greatest RMSE for all sites (Table 3, see Marianna, Jackson County, 2004; Cedara, 2005–2006; and Cathedral Peak, 2002). Cedara, in a mist-belt, for which the 2-h nowcast comparisons were for more than 15 months, and Cathedral Peak had an increased percentage of the number of days with *b* or *c* values <0 or *T*(*t*_sr − 2_) > *T*(*t*_sr − 4_) (Tables 3, Cedara, 2005–2006, and 4, Cedara, 2005–2006, and 3, Cathedral Peak, 2002, and 4, Cathedral Peak, 2002, respectively).

**Fig. 3:**
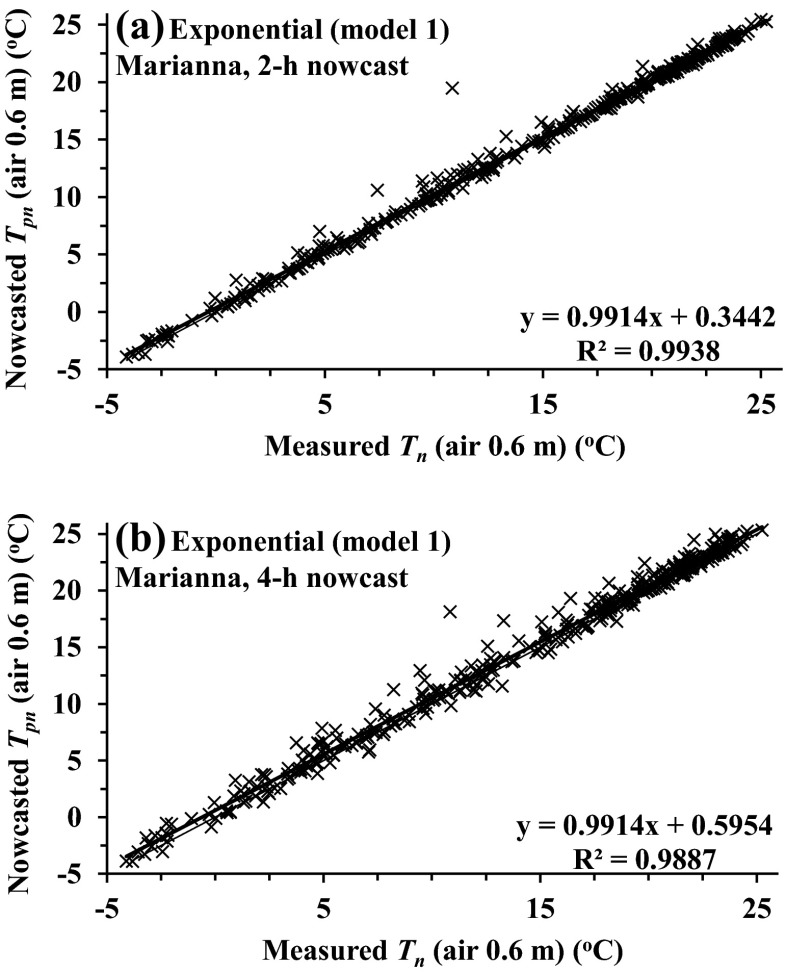
Regression plots for the experimental exponential model 1 for Marianna data for 0.6 m for **a** model-nowcasted *T*_pn_ for the 2-h-ahead nowcasted vs measured minimum *T*_*n*_ and **b** model-nowcasted *T*_pn_ for the 4-h-ahead nowcast vs measured minimum *T*_n_

**Fig. 4:**
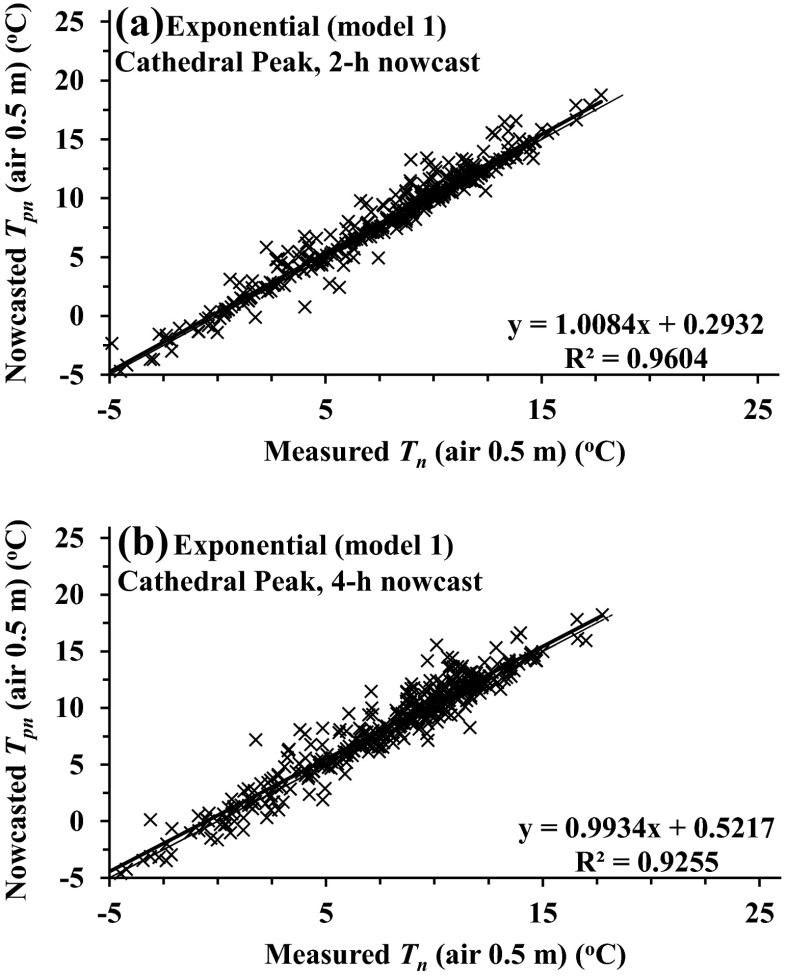
Regression plots for the experimental exponential model 1 for Cathedral Peak data for 0.5 m for **a** model-nowcasted *T*_pn_ for the 2-h-ahead nowcasted vs measured minimum *T*_n_ and **b** model-nowcasted *T*_pn_ for the 4-h-ahead nowcast vs measured minimum *T*_n_

Usually, application of model 1 for the sub-tropical sites yielded the best statistical comparisons with *T*_n_. The RMSEs for both exponential models 1 and 2 were consistently <1 °C for the 2-h nowcasts (Table 3, see Marianna, Jackson County, 2004; Cedara, 2005–2006; and Cathedral Peak, 2002). Models 3 and 4 based on the square root model were not as good for the 2-h nowcasts, with increased RMSE (Table 3, see Marianna, Jackson County, 2004; Cedara, 2005–2006; and Cathedral Peak, 2002).

For the sub-Saharan Kinsevere site, using 1-min air temperature measurements, all models performed well for the 2-h nowcasts (Table 3, see Kinsevere, Katanga province, DRC 2013–2014). Compared to the subtropical sites, the range in daily minimum air temperature for the Kinsevere dataset was much lower—about 5–19 °C. This resulted in larger intercepts compared to the subtropical sites. However, for the Kinsevere 4-h nowcasts, all models resulted in larger intercepts, slopes less than 1 and larger bias errors (Table 4, see Kinsevere, Katanga province, DRC 2013–2014).

The 4-h model nowcast comparisons, for all sites, often exhibited increased RMSE for all models—between about 15 and 80 % (Table 3, Marianna, Jackson County, 2004; Cedara, 2005–2006; and Cathedral Peak, 2002 compared to Table 4, Marianna, Jackson County, 2004; Cedara, 2005–2006; and Cathedral Peak, 2002, and Fig. 3a vs 3b and Fig 4a vs 4b).

### Implementation of the minimum temperature nowcast methodology into a near real-time web-based system

The model 1 and 2 (exponential) equations, using regression-determined *b* and *b* = 2.2, respectively, were added to the datalogger programme for calculating the nowcasted 2- and 4-h ahead air, grass and grass-surface temperatures. The results are displayed using the system described by Savage et al. (2014).

## Conclusions

Exponential and square root models for nowcasting the daily minimum air temperature for air, grass and grass-surface temperature 2 and 4 h before sunrise were successfully applied using sub-hourly temperature measurements from four subtropical sites with very different altitudes and from one central sub-Saharan site. Using the historical data, for both the 2- and 4-h nowcasts, model 1 (exponential) usually yielded the lowest RMSE. The modelled (*T*_pn_) vs measured (*T*_n_) comparisons for grass-surface and grass temperatures were more variable than those for air temperature with a significant increase in RMSE. For the nowcasts, in general, model 1 (exponential), for which the rate of reduction in nighttime temperatures was determined by covariance and variance instructions either in the datalogger (near real-time nowcasts) or in a spreadsheet using historic data, yielded the best statistical comparisons. For the sub-Saharan site, all models performed well for the 2-h nowcast. There was however large bias for the 4-h nowcasts. The display of near real-time data provided a convenient method for the display of the nowcasted minimum air, grass and grass-surface temperatures in a web-based system. The measurement and nowcasting system, as described, has potential economic benefit if implemented operationally.

## References

[CR1] Aberystwyth University (2014) Aberystwyth University—SimSphere. https://www.aber.ac.uk/en/iges/research-groups/earth-observation-laboratory/research/simsphere/. Accessed 10 May 2015

[CR2] Das HP, Doblas-Reyes FJ, Garcia A, Hansen J, Mariani L, Nain AS, Ramesh K, Rathore LS, Venkataraman S (2010). Weather and climate forecasts for agriculture. Guide to agricultural ,meteorological practices, Chap. 5.

[CR3] Garcia A, André R, de Melo-Abreu JP, Fereira RN, Prasad PVV, White D (2010). Climate and weather risk assessment for agricultural planning. Guide to agricultural meteorological practices, Chap. 7.

[CR4] Glickman TS (2000). Glossary of meteorology.

[CR5] Groen P (1947). Note on the theory of nocturnal radiational cooling of the earth’s surface. J Meteorol.

[CR6] Janiesch C, Matzner M, Müller O (2012). Beyond process monitoring: a proof-of-concept of event-driven business activity management. Bus Process Manag J.

[CR7] Johnson ME, Fitzpatrick EA (1977). A comparison of methods of estimating a mean diurnal temperature curve during the daylight hours. Arch Meteorol Geophys Bioklimatol Serv B.

[CR8] Lee S, Lee E, Park MS, Kwon BY, Kim H (2014). Short-term effect of temperature on daily emergency visits for acute myocardial infarction with threshold temperatures. PLoS ONE.

[CR9] National Oceanic and Atmospheric Administration (undated) Solar calculation details. http://www.esrl.noaa.gov/gmd/grad/solcalc/calcdetails.html Accessed 10 May 2015

[CR10] Parton WJ, Logan JA (1981). A model for diurnal variation in soil and air temperature. Agric Meteorol.

[CR11] Pelosi V (1986). Agrometeorologia: Leggi Fisiche per lo Studio del Microclima.

[CR12] Russo A, Coluccelli A (2006). Integration of a relocatable ocean model in the Mediterranean forecasting system. Ocean Sci Discuss.

[CR13] Savage MJ (2012). Estimation of frost occurrence and duration of frost for a short-grass surface. S Afr J Plant Soil.

[CR14] Savage MJ (2014) Web-based teaching, learning and research using real-time data from field-based agrometeorological measurement systems. MScAgric dissertation, University of KwaZulu-Natal, Pietermaritzburg, South Africa

[CR15] Savage MJ, Heilman JL (2009). Infrared calibration of net radiometers and infra red thermometers. Agric For Meteorol.

[CR16] Savage MJ, Abraha MG, Moyo NC, Babikir N (2014). Web-based teaching, learning and research using accessible real-time data obtained from field-based agrometeorological measurement systems. S Afr J Plant Soil.

[CR17] Snyder RL, de Melo-Abreu JP (2005). Frost protection: fundamentals, practices and economics.

[CR18] Wann M, Yen D, Gold HJ (1985). Evaluation and calibration of three models for daily cycle of air temperature. Agric For Meteorol.

[CR19] World Meteorological Organisation (2008) Guide to meteorological instruments and methods of observation. WMO, No. 8, 7th edn. Geneva, Switzerland

[CR20] Wu LY, Zhang JY, Dong WJ (2011). Vegetation effects on mean daily maximum and minimum surface air temperatures over China. Chin Sci Bull.

